# Inclusive fitness effects can select for cancer suppression into old age

**DOI:** 10.1098/rstb.2015.0160

**Published:** 2015-07-19

**Authors:** Joel S. Brown, C. Athena Aktipis

**Affiliations:** 1 Department of Biological Sciences, Cancer Center, University of Illinois at Chicago, Chicago, IL 60612, USA; 2 Cancer Biology and Evolution Program, Moffitt Cancer Center, Tampa, FL 33612, USA; 3 Department of Psychology, Center for Social Dynamics and Complexity, Center for Evolution and Medicine, Arizona State University, Tempe, AZ 85287-4501, USA; 4 Center for Evolution and Cancer, University of California San Francisco, San Francisco, CA 94143, USA

**Keywords:** parental investment, intergenerational transfers, cooperative breeding, grandparenting, inclusive fitness, Peto's paradox

## Abstract

Natural selection can favour health at youth or middle age (high reproductive value) over health at old age (low reproductive value). This means, all else being equal, selection for cancer suppression should dramatically drop after reproductive age. However, in species with significant parental investment, the capacity to enhance inclusive fitness may increase the reproductive value of older individuals or even those past reproductive age. Variation in parental investment levels could therefore contribute to variation in cancer susceptibility across species. In this article, we describe a simple model and framework for the evolution of cancer suppression with varying levels of parental investment and use this model to make testable predictions about variation in cancer suppression across species. This model can be extended to show that selection for cancer suppression is stronger in species with cooperative breeding systems and intergenerational transfers. We consider three cases that can select for cancer suppression into old age: (i) extended parental care that increases the survivorship of their offspring, (ii) grandparents contributing to higher fecundity of their children and (iii) cooperative breeding where helpers forgo reproduction or even survivorship to assist parents in having higher fecundity.

## 1. Introduction

There is more to Peto's paradox than just body size. As the papers in this volume and others suggest, low fecundity, low reproductive competitiveness [1], delayed reproduction and long reproductive lifespans favour the evolution of greater cancer suppression in multi-cellular organisms. But, this also raises the question of why many species have long post-reproductive cancer-free lifespans. Is continued cancer suppression post-reproductively merely cellular, physiological and immunological inertia; or can natural selection still favour cancer suppression into ‘old-age’? Here, we suggest ways, sometimes over-looked or unappreciated, by which selection for cancer suppression may be enhanced for younger and older age classes simultaneously.

We describe a simple model of the evolution of cancer suppression with varying levels of parental investment and other resource transfers to kin, and use this to make testable predictions about variation in cancer suppression across species based on levels of parental investment. This model suggests that social species with more intergenerational care (often altricial ones) may have more powerful cancer suppression mechanisms than comparable ones with little care (precocial species). We suggest that parental investment, intergenerational transfers, cooperative breeding and other inclusive fitness effects hold the key for how natural selection favours continued cancer suppression into older age classes including post-reproductives.

## 2. Model

Life-history theory tells us that populations at their stable age distributions will have an exponential growth rate, *r*, that can be derived from the Euler–Lotka equation [2,3]:2.1
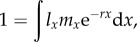
where *l_x_* is the probability of a newborn surviving until age *x*, *m_x_* is the expected number of newborns produced by an individual of age *x*, and *r* is the *per capita* growth rate of such a population with these survivorship and fecundity terms. The integral is evaluated from age 0 until the age beyond which no individuals survive. When population growth and fecundity are limited by females, then survivorship refers to females and offspring refer to daughters. The calculation of *per capita* growth rate may be a short- or long-term estimate depending upon the strength of density dependence or temporally auto-correlated stochastic effects. But, for our purposes, these issues are only indirectly relevant, not directly so.

Other useful life-history properties relevant to the discussion of the evolution of cancer suppression include net reproductive rate, age-specific reproductive value and generation time, respectively:2.2
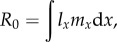
2.3
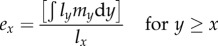
2.4
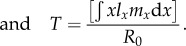


Following Brown *et al*. [4], the probability of surviving until age *x* is the product of surviving mortality from cancer, and surviving mortality from other causes. Furthermore, for a given cancer suppression trait that does not vary with age, the likelihood of dying from cancer between age *x* and *x* + 1 increases with age [5]. This is because time provides the opportunity to accumulate a sequence of cancer initiating mutations, as well as the time for an initiated cancer to grow, metastasize and result in death. This means that an increase in a cancer suppression trait will have smaller impacts early in life and compounded benefits later in life. This becomes particularly relevant when older individuals continue to reproduce and/or influence the fecundity or survivorship of offspring or relatives.

Maximal cancer suppression would evolve if such adaptations were cost free. Some of the costs associated with high levels of cancer suppression may include slower wound healing (because of lower rates of proliferation and cell motility), lower fertility (because of less invasive placentation) and other potential mechanisms and energetic costs that may divert resources from other somatic investments [1,6]. If cancer suppression diverts investment away from growth, reproduction and survivorship from other sources of mortality, then this leads to diminished returns from increased cancer suppression. If we assume that there are diminishing returns to increased cancer suppression adaptations—which there must be since age-specific survivorship from cancer must asymptote on one—and we assume that the costs of such adaptations accelerate in terms of missed fecundity and mortality from other sources, then there will be an evolutionarily stable level of cancer suppression, u*, that maximizes *per capita* growth given the other life-history properties of the organism.

Brown *et al*. [4] shows that cancer suppression as an adaptation increases with age at first reproduction (delayed reproduction), increasing fecundity with age, age at last reproduction and survivorship from other causes of mortality. Cancer suppression decreases in value with an increase in overall fecundity. In terms of life-history properties, cancer suppression should increase with longer generation time and when reproductive value increases or remains high with age. The direct effect of net reproductive rate on cancer adaptations is equivocal as it can increase or decrease through the front-loading or back-loading of survivorship and fecundity with age. So, an animal such as the meadow vole that has a life expectancy of less than 2 years and extremely high fecundity should have very low levels of cancer suppression adaptations. The converse is true for the similarly sized naked mole rat that has a very long reproductive life expectancy, low fecundity per female and an increase in reproductive value with age [7,8].

Once an individual is post-reproductive and *l_x_m_x_* = 0 for *x* > *x*′, selection no longer favours cancer suppression; whatever level of cancer suppression they possess has been sculpted by selection acting on all of the age classes *x* < *x*′. This does not mean that cancer becomes inevitable at this age, it just means selection cannot favour enhanced cancer suppression after *x*′ (i.e. the age of last reproduction). All else being equal, increasing *x*′ will select for higher levels of cancer suppression in the population.

However, selection can act to favour cancer suppression after old age if there are inclusive fitness effects by which relatives influence the survivorship or reproduction of their kin. This provides means for individuals to remain evolutionarily relevant beyond *x*′ and for net reproductive value to remain positive or even increase into old age. We shall discuss three of these scenarios, in turn, based on how they manifest in the life-history metrics that should influence the evolution of cancer suppression. They include: (i) extended parental care that increases the survivorship of their offspring, (ii) grandparents contributing to higher fecundity of their children, and (iii) cooperative breeding where helpers forgo reproduction or even survivorship to assist parents in having higher fecundity.

### (a) Extended parental care

The presence or absence of parents may be vital to the survival of the young. If parents after the first year or time period have only small effects on the survival of their offspring, then there will be little additional gain for reducing mortality from cancer via continuing parental contributions to offspring survivorship. Natural selection will favour even greater cancer suppression into older age classes if parents must survive several years into the future for their offspring to survive at all—namely *l_x_* = 0 for *x* ≤ *x*_c_ if the parents do not survive for at least *x*_c_ more years. This means that the probability of a newborn transitioning to the age when it is independent of parental care is discounted by the likelihood that the parents survive their next *x*_c_ years. For instance, for an offspring born to a parent of age *y*, its probability of surviving to age *x*_c_ will be discounted by (*l_y_*_+*x*c_/*l_y_*) relative to a species that has no or very little parental care. Finally, if the presence of parents, even post-reproductive ones, in the social group continues to increase the probability of their adult offspring's survival, then cancer suppression into old age will be adaptive. Parental investment will thus be expected to lead to enhanced selection for cancer suppression, but only when that investment occurs over an extended period of time.

Extended parental care plays an important role in survival of offspring for humans and also plays an important role in some non-human primates [9], a variety of other mammals [10], birds [11] and even crustaceans [12]. This model predicts that species such as these that have a long extent of post-reproductive offspring care and a strong influence of that care on offspring survival should have stronger selection on cancer suppression mechanisms. A promising pair of species could be something like the spotted hyaena [13], where matriarchs assist their daughters in the social hierarchy, versus brown hyaenas that are monogamous or polyandrous without social hierarchies [14]. The former species may reveal more cancer suppression than the latter. Other promising species that may exhibit unusually low cancer incidences into old age are those where ‘parents', ‘elders' and ‘grandparents' provide extended amounts of information transfer, experience and training to younger members of a social group (e.g. some dolphin species and whales).

### (b) Grandparent effects and inclusive fitness

Post-reproductive adults may not only enhance the survivorship of their offspring, adult or otherwise, but they may enhance the reproductive success of their offspring through care in the form of protection or resource transfers [15]. In this way, a female of age *x* may expect fecundity of *m_x_* whether her mother is alive or not, but this may increase to *m*′*_x_* = *m_x_* + *g* if the mother is still alive. In this way, *g*, is the additional fecundity effect of grandma on grandchildren via a daughter. For this effect to occur, both the mother-to-be and her mother must survive. The average age of an offpsring's mother is just the generation time, *T*. And so as an approximation, the net reproductive rate of a lineage is given by2.5
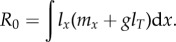
All else being equal, increasing *g*, decreasing *m_x_* and increasing the likelihood of surviving other causes of mortality will then select for greater cancer suppression and cancer suppression for older individuals even if they are post-reproductive [4]. This model therefore predicts greater selection for cancer suppression in species in which intergenerational transfers can enhance the survival of grandchildren. Grandparental care is found in species including humans, non-human primates, dolphins, pilot whales and some birds (as reviewed in [16]) and so higher levels of cancer suppression might be expected in those species.

Interestingly, this intergenerational effect on the advantages of cancer suppression might remain quite strong even if few females survive to see their grandchildren. For instance, if a grandparent can double her daughter's reproductive success, then *g* remains 20% of *m_x_* even if only say 10% survive to help their grandchildren—this may be relevant for humans where surviving to see one's grandchildren may have been historically small but consequentially large. Finally, the inclusive fitness effects of grandparents on the fecundity or survivorship of their adult offspring can be in addition to continued fecundity by the grandparents themselves (humans are among just a handful of species to have distinct menopause). Even if fecundity is constant with age, net reproductive value with age will generally decline as less of an adult's reproduction lies ahead than behind. In this way, older age classes will exert less selection for cancer suppression than younger. For such species, the positive intergenerational effects of older age classes on younger kin further selects against senescence and for cancer suppression into old age.

### (c) Cooperative breeding and helpers at the nest

In species like the acorn woodpecker, offspring even as young adults may remain in the parent's territory. While doing so, they may have increased survivorship from enjoying a safe, productive home range, and they generally choose to (or are coerced) into helping their parents raise their next set of siblings [17]. The net effect of this is to reduce fecundity at younger age classes and shift this fecundity to older age classes; in particular, it will have the effect of increasing the generation time if older adults have more offspring at the expense of younger adults. Both longer generation times and an increasing *m_x_* with age will select for increased cancer suppression that should manifest as lower cancer incidences for all age classes including continued legacy suppression for post-reproductives. Like the grandparent effect above, the helpers gain inclusive fitness benefits but this only happens if they and their parents are alive, and these offspring are born to the parents, thus increasing generation time as well as reproductive value with age. Unlike the grandparent effect, it is the direct increase in the fecundity of older individuals that drives increased cancer suppression. Thus, either assisting children to have more or better offspring, or having offspring enhance parental fitness should select for cancer suppression into old age.

Nature offers a number of taxonomically related combinations of species of similar body size but with different degrees of nest helpers that can be used to test some of the predictions of this model. For instance, Mexican jays and Florida scrub jays [18] have helpers, whereas this is less so for the Stellar's jay and blue jay. Woodpeckers offer examples of helper (acorn and red-cockaded woodpecker) and non-helper species (hairy and gila woodpecker). Marmots, a large rodent, provide a nice pair of species where the yellow-belly marmot has extensive female kin groups that assist in vigilance and collective protection of young [19], and the woodchuck that does not exhibit this sociality. And, perhaps the iconic example, is the naked molerat (only one female, and usually among the oldest, breeds while everyone else helps) that could be compared to several other molerat species that do not exhibit this extreme helping behaviour. In all of these cases, we expect greater cancer suppression into old age for the social system with helpers than for the species without helpers.

## 3. Discussion and conclusion

Our goal has been to describe how parental investment leads to selection for cancer suppression into old age or after reproductive age. We used life-history models to show that selection for cancer suppression is higher in species with cooperative breeding systems and intergenerational transfers. There are, however, a few caveats and limitations to note. Our model predicts that parental investment, grandparenting and cooperative breeding should lead to selection for greater cancer suppression into old age. This does not necessarily mean that species with these traits will have the lowest rates of cancer.

Factors contributing to cancer susceptibility including larger body size and longer lifespan may also correlate with those factors favouring strong cancer suppression mechanisms such as high parental investment, delayed reproduction and other slow life-history traits. Thus, closely related species that differ in parental investment, grandparental care and cooperative breeding provide some of the best means for testing this model's predictions. In this way, the model provides a framework for testing whether the cancer suppression patterns seen in humans and other species may partially be due to inclusive fitness effects. Future work integrating information about age of reproduction, lifespan, sociality and cancer incidence in diverse human societies and other species will be valuable for applying this model.

We have focused on the evolutionary pressures that can enhance cancer suppression in species with high parental investment and other forms of resource transfer to kin, but evolution could potentially also favour facultative cancer suppression based on current conditions. It remains an open question whether individual physiologies permit facultative calibration of cancer suppression to individual socio-ecological circumstances and age. Our model makes the following interesting prediction. If an individual ‘knows' it will be able to contribute to the well-being of kin long after reproductive age, then it should adjust physiological systems to allocate greater resources to cancer suppression. Such abilities to upregulate cancer suppression through facultative responses would have interesting implications for the viability of social and ecological interventions extending cancer-free life. Species that exhibit variation in lifespans and life-history strategies that vary with social status or ecotype would be the most logical candidates for testing this hypothesis.

Finally, our model connects cooperation theory and cancer biology in a novel way: it predicts that cooperation and resource transfers among kin may play a role in the selection for cancer suppression mechanisms at the organism level. Therefore, species that engage in higher levels of care, learning and kin-based cooperation are predicted to have greater selection for cancer suppression. This means that aspects of the social system and the nature of resource transfers in a species might actually have important effects on cancer susceptibility. It may even be the case that the evolution of cancer suppression mechanisms had important feedback effects on the evolution of social systems and kin-based cooperation. When bio-prospecting for cancer suppression mechanisms in wildlife, the predictions of this model can help guide future work in comparative oncology, especially for high-investing social organisms that may live long cancer-free lives after reproduction.
